# Joint Effects of Carbon Black Exposure and Dietary Antioxidant Vitamin Intake on Small Airway Dysfunction

**DOI:** 10.3389/fnut.2021.716398

**Published:** 2021-10-25

**Authors:** Tao Wang, Jianyu Li, Yi Liang, Wei Han, Jinglong Tang, Guo Cheng, Yuxin Zheng

**Affiliations:** ^1^Department of Occupational and Environmental Health, School of Public Health, Qingdao University, Qingdao, China; ^2^Laboratory of Molecular Translational Medicine, Key Laboratory of Birth Defects and Related Diseases of Women and Children (Sichuan University), Centre for Translational Medicine, Ministry of Education, West China Second University Hospital, Sichuan University, Chengdu, China; ^3^Department of Respiratory and Critical Care Medicine, School of Medicine, Qingdao Municipal Hospital, Qingdao University, Qingdao, China

**Keywords:** carbon black, air pollution, antioxidant vitamin, lung function, joint effect

## Abstract

**Objectives:** Small airway dysfunction is considered as a precursor of chronic obstructive pulmonary disease and asthma. Our aim was to explore the joint effects of carbon black (CB) exposure and antioxidant vitamin intake on small airway dysfunction.

**Methods:** A total of 70 CB packers (CBPs) and 107 non-CBPs were enrolled from an established cohort of CBP. Carbon content in airway macrophage (CCAM) quantified in induced sputum was used as a bio-effective dosimetry for exposure to CB. Logistic regression models were used to examine the odds ratios (ORs) of CB and dietary intake of antioxidant vitamins on small airway dysfunction, and the dose–response association.

**Results:** The prevalence of small airway dysfunction was 32.9% (23 of 70) among CBPs, and 19.6% (21 of 107) among non-CBPs. For each 2.72-fold increase in CCAM, the OR of small airway dysfunction was 2.31 (95% CI = 1.20–4.44). For every 10 mg day^−1^ increase of the vitamin C intake, the risk of small airway dysfunction decreased by 6% (OR = 0.94, 95% CI = 0.88–0.99). Compared to non-CB exposure and higher vitamin C intake, CB exposure and lower vitamin C intake (OR = 7.56, 95% CI = 1.80 to 31.81) were associated with an increased risk of small airway dysfunction.

**Conclusions:** Chronic exposure to a high level of CB aerosol increased the risk of small airway dysfunction in CB baggers. Dietary intake of vitamin C might be a modifiable factor for preventing small airway dysfunction.

## Introduction

Small airway dysfunction is considered as a precursor of chronic obstructive pulmonary disease (COPD) and asthma. More than 40% of Chinese adults aged 20 years or older had small airway dysfunction, accounting for more than 400 million people in China alone (1). However, evidence on risk and protective factors associated with small airway dysfunction is still limited. The nationally representative China Pulmonary Health (CPH) study showed that particulate matter (PM) with aerodynamic diameters <2.5 μm (PM_2.5_) increased the risk of small airway dysfunction (1).

The composition of airborne particles is complicated, and it is necessary to study the characteristics of primary particles. The fuel-derived PM in the inhalable size range is dominated by aggregates of nanoparticles of carbon black (CB), which is virtually pure elemental carbon (2, 3). Animal models have indicated that inhalation of CB could induce lung inflammation, fibrosis, emphysematous, epithelial hyperplasia, and lung carcinomas (2, 4–6). Our previous studies based on a cohort of carbon black packers (CBPs) with PM exposure levels over 800 μg/m^3^ also identified that exposure to CB was associated with reduced pulmonary function, Clara cell injury, higher levels of pro-inflammatory cytokine secretion, elevated permeability of acinar airways, and airway remodeling (7–10). However, the effect of CB exposure on small airway dysfunction was still unclear.

Furthermore, the possible modulatory effect of diet on lung health has been investigated in several studies. Dietary intake of various sources of antioxidants was associated with improvement of ventilatory function in adults (11–13). Elucidation of the associations between dietary nutrients and small airway dysfunction could provide a scientific basis for designing complex dietary supplements to alleviate the impact of air pollution on the small airway and prevent small airway diseases.

Therefore, we conducted a cross-sectional study from an acetylene black industry and non-exposed controls to test the hypothesis that (1) exposure to high levels of CB could induce small airway dysfunction in a dose-dependent manner, and (2) higher antioxidant vitamin (vitamins C and E, and β-carotene) intake could diminish the harmful effect of CB exposure on small airway dysfunction.

## Methods

### Study Population

Our CBP study was established in 2012 by recruiting CBPs from an acetylene black industry and non-CBP controls from a local water authority. Design details, including inclusion and exclusion criteria, have been reported elsewhere (7, 8). A follow-up visit was conducted in the fall of 2018, and spirometry was complete for 107 non-CBPs and 70 CBPs (14). Written informed consent from all participants was provided prior to the interview and any procedures. The CBP study was approved by the Medical Ethical Review Committee of the National Institute for Occupational Health and Poison Control, Chinese Center for Disease Control, and Prevention (protocol number: NIOHP201604).

### Exposure Assessment

We assessed PM_2.5_ and PM_2.5_-related elemental carbon in CB bagging facilities and control areas during the field visit in 2018. The technical details have been described in our previous studies (7, 10, 14).

### Carbon Content in Airway Macrophage Assay

Sputum collection, processing, slide preparation, and quality assessment were described in detail in our previous study (14). Upper quartile of the proportion of cytoplasm area occupied by carbon particles from 50 macrophages was used as the carbon content in airway macrophage (CCAM) index for all analyses (10, 14). CCAM, a quantitative measure for lung elemental carbon burden, serves as a bio-effective dosimeter for chronic exposure to CB aerosol and PM in air pollution (15, 16).

### Nutrient Intakes

The consumption of foods and food groups by the participants was collected by trained investigators *via* the validated 48-item food frequency questionnaire (FFQ) adapted to the Chinese population (17). It recorded the consumption of different foods (e.g., steamed bread, white rice, wheat noodle, bread, whole grain foods, potatoes, cakes, vegetables, fruits, dairy and dairy products, soybeans and its products, nuts, meat, eggs, fish and shrimp, and beverages) over the previous 12 months as frequencies. Dietary intake data were converted into nutrient intake data using the continuously updated in-house nutrient database, which was based on the China Food Composition (18). The antioxidant vitamin intakes (vitamins C and E, and β-carotene) were then calculated by multiplying the frequency of consumption of each food or beverage by the nutrient content of the portion and summing the intake of each nutrient for all items.

### Spirometry

Spirometry was conducted without inhaling bronchodilator by a certified technician using a portable calibrated electronic spirometer (CHESTGRAPH HI-701, Japan) in accordance with the American Thoracic Society and European Respiratory Society standards (19). Maximal mid-expiratory flow (MMEF), forced expiratory flow (FEF) at 50% of vital capacity, and FEF at 75% of vital capacity were used as indicators of lung function to estimate small airway dysfunction. Small airway dysfunction was diagnosed when at least two of the above three indicators were <65% of predicted values (1). This definition was comparable with that used in a previous study (1) in the Chinese population.

### Statistical Analysis

The associations between CB exposure status or CCAM and small airway dysfunction were assessed using logistic regression. Odds ratios (ORs) with 95% CI were applied. The first model (model 1) was unadjusted for potential confounding. The second model (model 2) adjusted for age, sex, body mass index, education level, family income level, smoking status, packyears, and drinking status. The interaction analyses, including cigarette smoking status × CB exposure, were designed *a priori*. In addition to the binary CB exposure status, CCAM was also used as a bio-effective dosimeter for exposure to CB aerosol in the lungs to characterize the dose response. Second, logistic regression models were used to examine the effect of dietary intake of antioxidant vitamins on small airway dysfunction, and the linear dose–response association. Antioxidant vitamins were treated as continuous variables in the preliminary analysis. To investigate the robustness of our results, we categorized the antioxidant vitamins into quartiles (Q1–Q4). Finally, we also estimated the joint effects representative of different combinations of high and low antioxidant vitamins intake and CB exposure. We stratified our subjects into four groups: non-CB exposure and higher vitamin C intake (≥median, 119 mg day^−1^) (reference group), non-CB exposure and lower vitamin C intake (< median, 119 mg day^−1^), CB exposure and higher vitamin C intake, and CB exposure and lower vitamin C intake, with the number of people in each group being 69, 37, 18, and 50, respectively.

All statistical analyses were conducted in 2020 using SAS 9.4 (SAS Institute Inc., Cary, NC, USA) and R 4.0.2 (R Foundation for Statistical Computing, Vienna, Austria). All *p-*values were two-tailed (α = 0.05).

## Results

### Exposure Assessment

**Figure 1:**
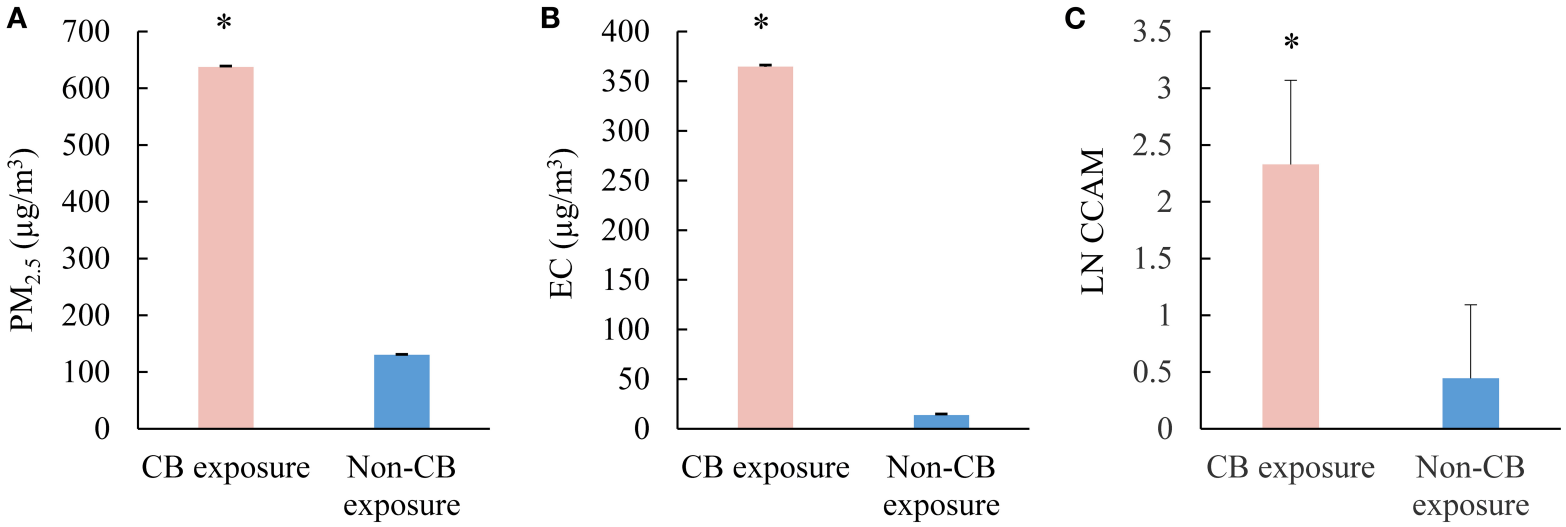
Exposure assessment of CB packers and non-CB packers. **(A)** Concentrations of PM_2.5_ in the work shop (μg/m^3^); **(B)** concentrations of elemental carbon in the work shop (μg/m^3)^; **(C)** the levels of CCAM in the participants. CB, carbon black; PM_2.5_, particulate matter with aerodynamic diameters <2.5 μm; LN CCAM, natural log transformed carbon content in airway macrophage. *p < 0.05.

Geometric means of PM_2.5_ and elemental carbon in CB bagging areas were 637.4 ± 1.7 and 364.6 ± 1.7 μg/m^3^, which were significantly higher than that seen in the reference areas (130.0 ± 1.2 and 13.8 ± 1.2 μg/m^3^, Figures 1A,B). CCAM in CBPs was significantly higher than that seen in non-CBPs (Figure 1C).

### Characteristics of Study Subjects

Age and body mass index (BMI) were comparable between the CBP and non-CBP groups. Compared with non-CBPs, CBPs were more likely to be men and ever smokers, and to report less packyears among ever smokers (Table 1). CBPs had lower FVC, FEV_1_, MMEF, and FEF_50%_ than non-CBPs. Overall, the prevalence of small airway dysfunction was 24.9% (44 of 177) in the study population (Table 1), and none had FEV_1_/FVC ratio <70%.

**Table 1:** General characteristics in the study population (*n* = 177).

**Variable**	**CBP (*n* = 70)**	**Non-CBP (*n* = 107)**	***P***
**Demographics**			
Age (year, $\bar{x}$ ± S)	48.91 ± 4.24	48.91 ± 4.30	0.991^a^
Male (*n*, %)	63 (90.00)	58 (54.21)	<0.001^b^
<12th grade (*n*, %)	67 (95.71)	43 (40.57)	<0.001^b^
Ever smokers (*n*, %)	49 (70.00)	48 (44.86)	0.001^b^
Packyears in ever smokers (M, P_25_-P_75_)	15.00 (7.50–22.50)	21.75 (13.00–32.25)	0.003^c^
Current alcohol user (*n*, %)	52 (75.36)	78 (73.58)	0.793^b^
Height (cm, $\bar{x}$ ± S)	166.47 ± 6.43	166.64 ± 7.87	0.882^a^
Weight (kg, $\bar{x}$ ± S)	70.77 ± 12.11	66.81 ± 12.37	0.037^a^
BMI (kg/m^2^, $\bar{x}$ ± S)	25.71 ± 3.58	24.64 ± 3.74	0.062^a^
**Spirometry**			
FVC % predicted (%, $\bar{x}$ ± S)	91.37 ± 11.74	100.97 ± 11.98	<0.001^a^
FEV_1_ % predicted (%, $\bar{x}$ ± S)	92.14 ± 12.68	99.10 ± 11.44	<0.001^a^
FEV_1_/FVC % predicted (%, $\bar{x}$ ± S)	115.93 ± 8.37	113.50 ± 8.58	0.065^a^
MMEF % predicted (%, $\bar{x}$ ± S)	82.89 ± 23.11	93.96 ± 23.89	0.003^a^
FEF_50_ % predicted (%, $\bar{x}$ ± S)	77.61 ± 22.76	86.37 ± 23.92	0.016^a^
FEF_75_ % predicted (%, $\bar{x}$ ± S)	67.77 ± 24.09	76.04 ± 29.55	0.052^a^
Small airway dysfunction (*n*, %)	21 (19.63%)	23 (32.86%)	0.052^a^

*CBP, carbon black packer; $\bar{x}$, mean; M, median; P_25_, 25th percentile; P_75_, 75th percentile; BMI, body mass index; FVC, forced vital capacity; FEV_1_, forced expiratory volume in 1 s; FEV_1_/FVC, ratio of the first second forced expiratory volume to forced vital capacity; MMEF, maximal mid-expiratory flow; FEF, forced expiratory flow*.

a *Student t-test*.

b *Chi-square test*.

c *Wilcoxon rank sum test*.

### Effect of Carbon Black Exposure on Small Airway Dysfunction

The prevalence of small airway dysfunction was 32.9% (23 of 70) among the CBPs, and 19.6% (21 of 107) among non-CBPs. Compared with non-CBPs, the CBPs were significantly associated with increased risk of small airway dysfunction, with an adjusted OR of 3.83 (95% CI = 1.21–12.10, Table 2). The effect of CB exposure on small airway dysfunction did not vary by smoking status (*p*_interaction_ = 0.608), suggesting no potential confounding effects of cigarette smoking.

**Table 2:** Effects of CB exposure on small airway dysfunction.

**Variable**	**Model 1^a^**		**Model 2^b^**	
	**OR (95% CI)**	***p***	**OR (95% CI)**	***p***
CBP vs. non-CBP	2.00 (1.01, 4.00)	0.048	3.83 (1.21, 12.10)	0.022
LN CCAM	1.47 (0.99, 2.19)	0.055	2.31 (1.20, 4.44)	0.012

*CBP, carbon black packer; OR, odd ratio; LN CCAM, natural log transformed carbon content in airway macrophage*.

a *Model 1 was unadjusted*.

b *Model 2 was adjusted for age, sex, body mass index, education level, family income level, smoking status, packyears, and drinking status*.

Because CCAM distribution was skewed, log-transformed CCAM was used to quantify its association with small airway dysfunction in the dose–response analysis. For each 2.72-fold increase in CCAM, the OR of small airway dysfunction was 2.31 (95% CI = 1.20–4.44, Table 2). In addition, the association between CCAM and small airway dysfunction did not vary by smoking status (*p*_interaction_ = 0.635).

### Effect of Dietary Intake of Antioxidant Vitamins on Small Airway Dysfunction

**Figure 2:**
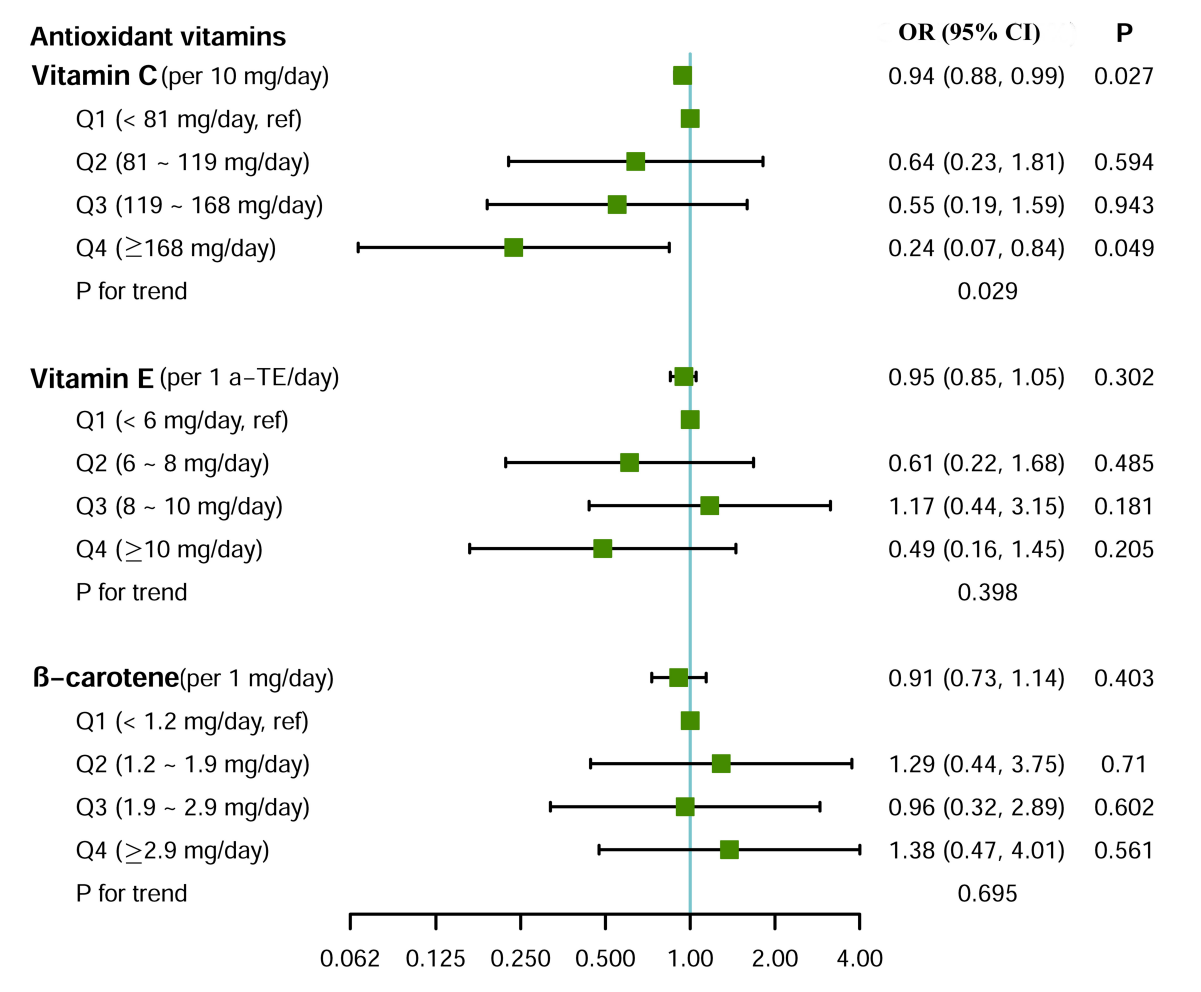
Effect of dietary intake of antioxidant vitamins on small airway dysfunction. Logistic regression model adjusted for age, sex, body mass index, education level, family income level, smoking status, packyears, and drinking status. For the test of trend, we calculated the association with small airway dysfunction by treating the categories of antioxidant vitamin as ordinal variables.

Figure 2 shows the associations between dietary intake of the antioxidant vitamins and small airway dysfunction. After adjusting for potential covariates, for every 10 mg day^−1^ increase of the vitamin C intake, the risk of small airway dysfunction decreased by 6% (OR = 0.94, 95% CI = 0.88–0.99). A similar dose–response pattern was observed when study subjects were divided equally into four groups based on vitamin C intake levels (*p*_fortrend_ = 0.029, Figure 2). There were no statistically significant associations between vitamin E, β-carotene intake, and small airway dysfunction in the univariate or adjusted models.

### Joint Effects of Carbon Black Exposure and Vitamin C Intake on Small Airway Dysfunction

**Figure 3:**
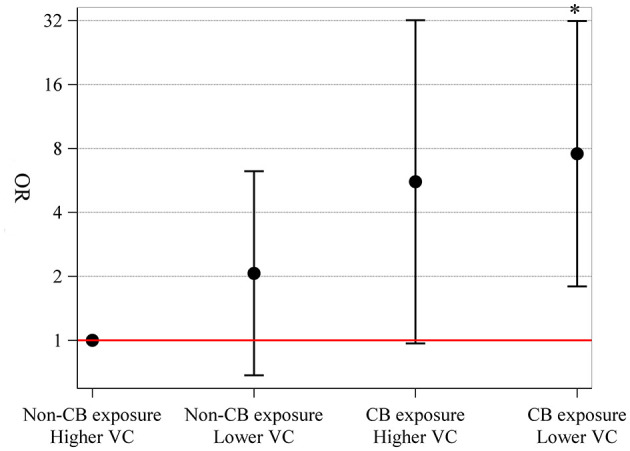
Joint effects of CB exposure and vitamin C intake on small airway dysfunction. We stratified our subjects into four groups: non-CB exposure and higher vitamin C intake (≥119 mg day^−1^) (reference), non-CB exposure and lower vitamin C intake (<119 mg day^−1^), CB exposure and higher vitamin C intake, and CB exposure and lower vitamin C intake, with the number of people in each group being 69, 37, 18, and 50, respectively. Joint effects were explored using logistic regression model with adjustment for age, sex, body mass index, education level, family income level, smoking status, packyears, and drinking status. CB, carbon black; VC, vitamin C.

Compared to non-CB exposure and higher vitamin C intake, non-CB exposure and lower vitamin C intake (OR = 2.07, 95% CI = 0.68–6.25), CB exposure and higher vitamin C intake (OR = 5.57, 95% CI = 0.97–32.08), and CB exposure and lower vitamin C intake (OR = 7.56, 95% CI = 1.80–31.81) were associated with an increased risk of small airway dysfunction (Figure 3). However, there was no evidence of interaction of CB exposure and vitamin C intake on small airway dysfunction (*p* = 0.630).

## Discussion

Our study indicated that chronic exposure to a high level of CB aerosol increased the risk of small airway dysfunction in CB baggers. Moreover, we confirmed a linear dose–response relationship between CCAM and small airway dysfunction, independent of smoking status. Most importantly, dietary intake of vitamin C was a modifiable factor for preventing small airway dysfunction.

The adverse pulmonary effects of chronic CB exposure have been explored in previous research. Persistent CB exposure was significantly associated with an increased risk of respiratory symptoms and restrictive ventilator disorders, and obviously reduced lung function (20–22). Our previous studies also found that the exposure to CB was associated with a significant reduction in FEV_1_% and FEV_1_/FVC (7, 8), but no workers had met spirometry diagnosis of airway obstruction (i.e., FEV_1_/FVC < 0.70) yet (10). In this study, according to the spirometric diagnostic criteria of China Pulmonary Health study (1), the prevalence of small airway dysfunction in the CBP group (32.9%) was significantly higher than that in the non-CBP group (19.6%). The results suggested that small airway dysfunction might be an early lung injury caused by chronic exposure to nano-scale CB aerosol and emerged prior to the occurrence of large airway diseases or emphysematous changes in this relatively young group of study subjects. However, as a cross-sectional study, the contribution of small airway dysfunction to CB exposure induced chronic lung diseases such as COPD, and the reversibility of observed changes could not be delineated. Furthermore, we used CCAM to assess the lung dose of CB in each worker and identified a linear dose–response with small airway dysfunction, which supported that CCAM as a good bio-effective biomarker that could bridge CB inhalation exposure and lung effects.

Regarding diet, there was evidence of relationships between certain micronutrients, especially those with antioxidant properties, and lung function (11). An analysis of the Third National Health and Nutrition Examination Survey (NHANES III) of the U.S. population demonstrated that increased intake of vitamin E, vitamin C, and total carotenes were all positively associated with FEV_1_ (23). A longitudinal study based on the European Community Respiratory Health Survey (ECRHS) showed that vitamin C was associated with a slower FVC decline over the 10-year follow-up (12). Vitamin C has previously been linked with higher lung function in several studies using different measures of intake, including dietary recall and plasma vitamin C concentrations (11). However, evidence was less consistent for other antioxidants. The Veterans Administration Normative Aging Study (NAS) have shown that vitamin E intake was positively associated with both FEV_1_ and FVC (24), whereas randomized controlled trials (RCTs) revealed no effect of β-carotene (25) or vitamin E (26) on lung function. Similarly, we demonstrated that higher amounts of vitamin C intake were associated with a lower risk of small airway dysfunction and found no evidence of an association between small airway dysfunction and intake of vitamin E or β-carotene.

In addition to demonstrating an association between vitamin C intake and small airway dysfunction, our results suggested that vitamin C intake might reduce the adverse effect of CB exposure on small airway dysfunction. Emerging data supported a role for mitigation of air pollution health risks *via* nutrition in a range of chronic diseases (27, 28). Vitamins C and E appeared to modify the short-term effects of PM_10_ on asthma and COPD exacerbations (29). In Mexico City, a cohort study concluded that dietary vitamin C intake provided some protection against the effect of ozone on the pulmonary function of asthmatic children (30). Furthermore, the Recommend Dietary Allowance (RDA) of vitamin C for adult men (aged 19 and older) is 90 mg day^−1^ (31). Our result indicated that a significant protective effect was only found in the highest category of vitamin C intake (≥168 mg day^−1^). Therefore, the RDA of vitamin C for CB workers might be increased to reduce the risk of small airway dysfunction. Future studies with a large sample size are warranted to confirm the RDA of vitamin C for CB workers.

The lungs exist in a high-oxygen environment, and exposures and inflammation can increase the burden of oxidants further (11). The balance between these potentially toxic substances and the protective actions of antioxidant defenses, including those derived from the diet, might play an important role in the pathogenesis of lung disease and loss of lung function over time (11, 32). Vitamin C was an exogenous non-enzymatic antioxidant that participated in the primary lung defense against reactive oxygen species (30). Vitamin C as a cofactor for enzymes (e.g., prolyl hydroxylases) could directly neutralize free radicals and suppress macrophage secretion of superoxide anions *in vitro* (33). It had very high redox potential and could reduce the most reactive oxygen species present in human tissues. However, fat-soluble vitamins, such as vitamins E and β-carotene, were hydrophobic and required dietary lipids to be absorbed. For example, when fats were present in the diet, vitamin E could be packaged into chylomicrons and transported in circulation. Therefore, the fat content of the diet could have a significant impact on the absorption of such nutrients.

Taken together, these results suggested that micronutrient status might impact small airway, and that nutrition interventions could be useful in a public health campaign aimed at the prevention of small airway dysfunction. In the future, well-designed interventional trials are needed to confirm these associations and to establish whether dietary interventions are effective in the prevention of small airway dysfunction. Population at high risk of small airway dysfunction, such as the CBPs, might be a target population that would incur the most benefit from the results of such trials. The low cost and high safety of dietary interventions, such as counseling to increase fruit and vegetable intake to maintain or improve lung function, make this a very attractive intervention for clinicians working with populations of patients either with or at risk for small airway dysfunction. Deficits in small airways might predispose certain individuals to an earlier diagnosis of impaired lung function; therefore, interventions to prevent small airway dysfunction are of interest.

Although ambient exposures were widely characterized and often based on modeling or predicted exposures, studies examining the effect of CB exposure on lung health were less prevalent. The CB exposure was pure elemental carbon and provided distinct but complementary evidence to studies of PM exposures, representing a strength of the current study. Identification of one modifiable risk factor (dietary intake) for small airway dysfunction in this population was another major strength. CCAM assessed as the accurate assessment of personal CB exposure was also the strength in this study.

The limitations of this study should also be considered. First, investigations of the association between single nutrients and disease might not accurately reflect a specific dietary habit or dietary behavior, as neither foods nor nutrients were consumed in isolation. Second, due to the small sample size in this study, adjustment for many potential confounding factors may reduce the power of the study and increase the margin of error, while further studies with large sample size are warranted in the future. Lastly, only high vitamin C intake may exert a protective effect, and an intervention trial will be needed to confirm these effects.

## Conclusions

In summary, we presented results from a cohort of CBP demonstrating the association between CB exposure (harmful), vitamin C intake (protective), and small airway dysfunction. Higher vitamin C intake was associated with diminished harmful effect of CB exposure on small airway dysfunction.

## Data Availability Statement

The raw data supporting the conclusions of this article will be made available by the authors, without undue reservation.

## Ethics Statement

The studies involving human participants were reviewed and approved by Chinese Center for Disease Control and Prevention. The patients/participants provided their written informed consent to participate in this study.

## Author Contributions

TW, WH, and JT contributed to the conceptualization of the study. TW, JL, and YL involved in the methodology of the study and performed formal analysis. TW, JL, and JT executed investigation. TW contributed to data curation and writing—original draft preparation. GC and YZ involved in writing—review and editing and in supervision. YZ helped in funding acquisition. All authors have read and agreed to the published version of the manuscript.

## Funding

This study was supported by the National Key Research and Development Program of China (Grant No. SQ2017YFC1600200) and National Natural Science Foundation of China (Grant No. 91943301). The study funders had no role in the design and conduct of the study, collection, management, analysis, and interpretation of the data, preparation, review or approval of the manuscript, or decision to submit the manuscript for publication.

## Conflict of Interest

The authors declare that the research was conducted in the absence of any commercial or financial relationships that could be construed as a potential conflict of interest.

## Publisher's Note

All claims expressed in this article are solely those of the authors and do not necessarily represent those of their affiliated organizations, or those of the publisher, the editors and the reviewers. Any product that may be evaluated in this article, or claim that may be made by its manufacturer, is not guaranteed or endorsed by the publisher.
